# Sildenafil Counteracts the In Vitro Activation of CXCL-9, CXCL-10 and CXCL-11/CXCR3 Axis Induced by Reactive Oxygen Species in Scleroderma Fibroblasts

**DOI:** 10.3390/biology10060491

**Published:** 2021-05-31

**Authors:** Cristina Antinozzi, Paolo Sgrò, Francesco Marampon, Daniela Caporossi, Francesco Del Galdo, Ivan Dimauro, Luigi Di Luigi

**Affiliations:** 1 Unit of Endocrinology, Department of Movement, Human and Health Sciences, University of Rome “Foro Italico”, 00135 Rome, Italy; paolo.sgro@uniroma4.it (P.S.); francesco.marampon@uniroma1.it (F.M.); luigi.diluigi@uniroma4.it (L.D.L.); 2 Department of Radiotherapy, Sapienza University of Rome, 00185 Rome, Italy; 3 Unit of Biology and Genetic, Department of Movement, Human and Health Sciences, University of Rome “Foro Italico”, 00135 Rome, Italy; daniela.caporossi@uniroma4.it (D.C.); ivan.dimauro@uniroma4.it (I.D.); 4 Leeds Institue of Rheumatic and Musculoskeletal Medicine and Diseases and NIHR Biomedical Research Centre, University of Leeds, Leeds LS2 9JT, UK; F.DelGaldo@leeds.ac.uk

**Keywords:** chemokines, systemic sclerosis, reactive oxygen species, fibrosis, sildenafil

## Abstract

**Simple Summary:**

Systemic sclerosis (SSc) is a complex autoimmune disease characterized by different clinical features and a high risk of mortality due to multi-organ fibrosis. Oxidative stress plays a key role in SSc pathogenesis, and an altered redox state could be responsible for abnormal inflammatory condition, tissue damage and fibrosis. Different pro-inflammatory mediators (cytokines and chemokines) occur from the earlier to the last stage of this disease, but their relationship with clinical findings is still unclear. To this purpose, studies are still required which work to find biomarkers mirroring the disease progression and potential pharmacological targets improving treatment response. In this study, we demonstrated that the vasoactive drug sildenafil, a phosphodiesterase type 5 inhibitor commonly used to treat pulmonary hypertension, reduces the expression and secretion of pro-inflammatory chemokines in dermal fibroblasts isolated from SSc patients exposed to reactive oxygen species. We demonstrated that this effect relies on the ability of sildenafil to interfere with the pro-inflammatory pathways involved in the expression of these molecules and its capacity to counteract the plasma membrane translocation of their specific receptor. These results sustain clinical studies to consider the use of sildenafil in preventing tissue damage and fibrosis in systemic sclerosis by targeting essential biomarkers of disease progression and reducing the oxidative-stress-induced inflammation.

**Abstract:**

Oxidative stress plays a key role in systemic sclerosis (SSc) pathogenesis, and an altered redox homeostasis might be responsible for abnormal inflammatory status, fibrosis and tissue damage extension. In this study, we explored the effect of the phosphodiesterase type 5 inhibitor sildenafil in modulating the activation of the CXCL-9, -10, -11/CXCR3 axis, which is fundamental in the perpetuation of inflammation in different autoimmune diseases, in the cell culture of SSc human dermal fibroblasts exposed to a pro-oxidant environment. We observed that sildenafil significantly reduced gene expression and release of CXCL-9, -10 and -11, inhibited the CXCR3 action and suppressed the activation of STAT1-, JNK- and p38MAPK pathways. This in vitro study on dermal fibroblasts supports clinical studies to consider the efficacy of sildenafil in preventing tissue damage and fibrosis in SSc by targeting central biomarkers of disease progression, vascular injuries and fibrosis and reducing the pro-inflammatory activation induced by oxidative stress.

## 1. Introduction

Systemic sclerosis (SSc), or scleroderma, is an autoimmune disease characterized by a progressive and systemic multi-organ fibrosis, as well as by abnormal immune system activation, the production of immunological mediators, and increased extracellular matrix deposition [1]. The disease shows a multifactorial etiology involving immune system alterations, infectious agents, as well as genetic and environmental factors. Despite the improvement of clinical outcomes, the mechanisms responsible for the disease maintenance and progression remain largely unknown [2,3,4]. To date, it is still a priority to identify potential therapeutic targets that could make this disease treatable [1,5].

Several studies suggest the central role of oxidative stress in SSc pathogenesis and demonstrate that an altered redox state could be crucial since it is responsible for abnormal pro-inflammatory and pro-fibrotic pathways initiation and tissue damage extension [6,7]. Moreover, it is generally accepted that the activation and transformation of fibroblasts is at least partly a downstream effect of the perturbed immune response [8,9,10]. This is associated with an enhanced secretion of inflammatory mediators such as CXC chemokines, including CXCL-9 (MIG), CXCL-10 (IP-10) and CXCL-11 (I-TAC/IP9), which are known to play a pivotal role in SSc pathogenesis [11,12,13,14,15]. These chemokines work in binding the CXC motif receptor 3 (CXCR3), thus polarizing the migration of CXCR3-positive immune cells and amplifying the inflammatory responses that lead to tissue damage and clinical manifestations [3,16]. Furthermore, these chemokines play a critical role in mediating oxidative-stress-induced inflammatory response [17,18], and they may act in SSc as biomarkers for disease progression, fibrosis, and micro-vascular abnormalities [13].

The complexity of SSc treatment results from the need to inhibit the autoimmune process, inflammation and organ-specific involvement. To date, therapeutic decisions should be made after an appropriate assessment of symptoms, disease duration, activity and complications.

The phosphodiesterase type 5 inhibitor (PDE5i) sildenafil is one of the commonly used drugs in the management of pulmonary hypertension secondary to SSc and vascular damages [19,20,21,22,23,24]. In the past, several authors have also investigated the potential effects of this molecule in the treatment of skin involvement; however, no encouraging outcomes are available, and as such, further results validated in a larger controlled study are needed.

Epidemiological observations indicate that patients with Raynaud’s phenomenon and antinuclear antibodies (ANAs) are at high risk of developing SSc. Thus, the dissection of the mechanisms underlying the PDE5i-induced modulation of pro-inflammatory and pro-fibrotic cytokines following reactive oxygen species (ROS) may pave the way for extending the scope of treatment with sildenafil in patients at risk of developing SSc and also in patients at the last stages of the disease when skin fibrosis occurs.

Recently, we have demonstrated that sildenafil could counteract the detrimental effects of the pro-oxidant environment in SSc fibroblasts by reducing the production and release of inflammatory mediators, thereby protecting cells from genotoxic damage [25,26]. In addition, we documented that sildenafil can specifically target human skeletal muscle cells that are challenged by inflammatory stimuli, thereby reducing CXCL10 release in association with the impairment of some intracellular paths activated by the inflammatory milieu [3].

The aim of this study was to evaluate the ability of sildenafil to modulate gene expression and the release of chemokines CXCL-9, CXCL-10 and CXCL-11. Moreover, we evaluated the capacity of sildenafil to affect CXCR3 cellular localization and the transduction pathway responsible for chemokines expression and release.

## 2. Materials and Methods

### 2.1. Chemicals

Cell culture medium DMEM/Ham’s F-12, phosphate-buffered saline Ca^2+^/Mg^2+^-free, trypsin, bovine serum albumin (BSA), antibiotics, hydrogen peroxide (H_2_O_2_), phosphodiesterase type 5 inhibitor (PDE5i) sildenafil citrate salts (S) (98%) and secondary antibodies were purchased from Sigma Aldrich (Merck KGaA, Darmstadt, Germany). The reagents for SDS-PAGE were from Santa Cruz (Santa Cruz, CA, USA) and Cell Signaling (Leiden, The Netherlands). For RNA extraction, TRIzol RNA isolation reagent was purchased from Ambion™. For reverse transcription, 10 mM dNTP Mix, Random Primers, RNaseOUTNAse™ Ribonuclease inhibitor, DNase I^®^ and SuperScript^®^ III Reverse were purchased from Invitrogen. SYBR^®^ Green PCR Master Mix for qPCR was purchased from Bio-Rad Laboratories, Inc. (Hercules, CA, USA). Plasticware for cell cultures and disposable filtration units for growth media preparation were purchased from Corning (Milan, Italy).

### 2.2. Cell Cultures and Treatments

Human dermal fibroblasts derived from excisional skin biopsies from 3 patients with early diffuse cutaneous SSc (dcSSc) and 3 healthy controls were isolated at the SSc clinic at Leeds Institute of Rheumatic and Musculoskeletal Medicine (UK) and processed as previously described [27]. Informed consent was obtained and approved by the National Research Ethics Service Committee (REC 10/H1306/88). Cells were treated for 1 h (h) or 24 h with H_2_O_2_ (100 μM) in the presence or absence of a pre-treatment of 30 min with sildenafil (1 μM). H_2_O_2_ concentration was selected according to the literature [25,26] to induce cytokine secretion by SSc cells. Sildenafil concentration was selected on the basis of the near-therapeutic doses used to treat erectile dysfunction, according to its pharmacokinetics (Cmax and area under the time–concentration curves, AUC). All experiments were performed using an equal number of cells obtained from 3 dcSSc and 3 healthy control patients.

### 2.3. Cytokine Secretion Assay

Healthy and SSc fibroblasts were plated at 4 × 10^4^ cells/mL in a 96-well tissue culture plate and treated for 24 h with H_2_O_2_ (100 μM) in the presence or absence of a pre-treatment of 30 min with sildenafil (1 μM). Cell culture supernatants were analyzed for CXCL-9, CXCL-10 and CXCL-11 by magnetic-bead-based multiplex assay according to the manufacturer’s protocol, as previously described [25,26]. Data acquisition and analysis were performed respectively by Bio-Plex 200 System™ and Bio-Plex Manager™ 6.0 software (Bio-Rad, Hercules, CA, USA). Experiments were performed in triplicate.

### 2.4. RNA Extraction, Reverse Transcription and Real-Time Quantitative PCR

Total RNA was obtained from ≈4.0 × 10^4^ SSc fibroblasts treated for 24 h with H_2_O_2_ (100 μM) in the presence or absence of a pre-treatment of 30 min with sildenafil (1 μM) using TRIzol according to the manufacturer’s instructions and as previously described [28]. cDNA was obtained by the reverse transcription of 1 μg of total RNA. RT-qPCRs were performed as previously described [25]. Fluorescence intensities were analyzed using the manufacturer’s software (7500 Software v2.05), and relative amounts were evaluated using the 2^−∆Ct^ method and normalized for β-actin. Data are expressed as fold increase. Sequences of primers for RT-PCR analysis are shown in Table 1.

### 2.5. Protein Content Analysis

Healthy and SSc fibroblasts were pre-treated with sildenafil 1 μM or vehicle and then stimulated for 1 h with 100 μM H_2_O_2_. After 1 h, cells were harvested and lysed in RIPA buffer (150 mM NaCl, 50 mM Tris-HCl pH8, 1 mM EDTA, 1% NP40, 0.25% sodium deoxycholate, 0.1% SDS, water to volume), supplemented with protease and phosphatase inhibitor cocktails. The immunoblot analysis was performed as previously described [29,30]. In particular, an equal amount of proteins (20–30 μg) was resolved in SDS-polyacrylamide (Bio-Rad, Hercules, CA, USA) gels (10–12%) and transferred onto nitrocellulose membranes (Amersham). Membranes were incubated with primary and secondary antibodies diluted in Tween Tris-buffered saline (TTBS). Proteins were revealed by the enhanced chemiluminescence system (ECL plus; Millipore, Burlington, MA, USA). Image acquisition was performed with Image Quant Las 4000 software (GE Healthcare) and densitometric analysis with Quantity One^®^ software (Bio-Rad, Hercules, CA, USA). The antibodies utilized were p-STAT1 (Tyr 701), total STAT1, p-P38MAPK (Thr180/Tyr182) and total P38MAPK by Cell Signaling; p-JNK (Thr183/Tyr185) and total JNK by Millipore; and phospho-alpha B-crystallin (Ser59) (p-CRYAB) and alpha B-crystallin (CRYAB) by Enzo Life Sciences (Milan, Italy).

### 2.6. Immunofluorescence Analysis

Immunofluorescence analysis was performed as previously described [31,32]. For CXCR3 plasma membrane localization, 10^4^ cells were seeded onto glass coverslips and maintained for 24 h in growth medium. After 24 h, cells were pre-treated with sildenafil 1 μM or vehicle and then stimulated for 1 h with 100 μM H_2_O_2_. To evaluate CXCR3 in the plasma membrane, cells were fixed with 4% paraformaldehyde (PFA) and incubated with blocking buffer containing 3% BSA for 30 min at room temperature. AbI CXCR3 (Santa Cruz, 1:100) was incubated overnight, followed by Cy3-conjugated secondary Ab (1:1000). For method specificity, slides lacking the primary Abs were processed. DAPI nucleic acid stain was used to stain nuclei (1:10,000). Images were acquired at 100× magnification, and slides were examined with Zeiss Z1 microscope and Leica TCS SP2 (Leica, Milano, Italy). Experiments were performed three times with different cell preparations.

### 2.7. Statistical Analysis

For data generation, experimental triplicates were performed. All data were represented as mean ± standard error of the mean (SEM) or as fold increase vs. untreated cells or H_2_O_2_-treated cells. Protein and mRNA content were analyzed by Mann–Whitney *t*-test analysis, one- or two-way ANOVA with Bonferroni’s correction, where necessary. GraphPad Prism 8.0 (La Jolla, CA, USA) was used for all statistical analyses. A *p*-value < 0.05 was considered significant.

## 3. Results

### 3.1. Sildenafil Reduced CXCL-9, CXCL-10 and CXCL-11 Secretion Induced by Hydrogen Peroxide in SSc Fibroblasts

**Figure 1:**
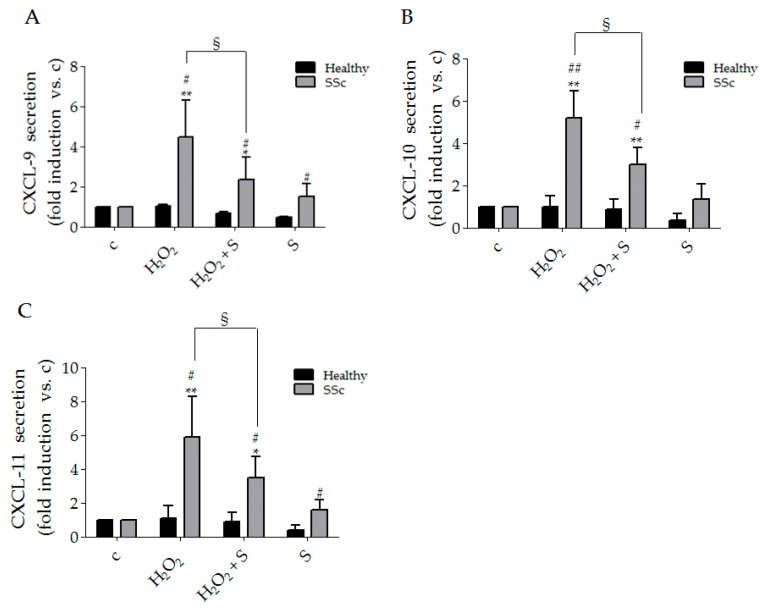
Supernatants from human healthy (black columns) and SSc (grey columns) fibroblasts cultures exposed to H_2_O_2_ (100 μM, 24 h) in the presence or absence of sildenafil (1 μM). Cells were analyzed for CXCL-9 (**A**), CXCL-10 (**B**) and CXCL-11 (**C**) content. Data are presented as the fold increase vs. control taken as 1 ± SEM (*n* = 3). Statistical significance was determined using ANOVA with Bonferroni’s post-hoc test. * *p* < 0.05 and ** *p* < 0.01 vs. relative control within group; ^#^ *p* < 0.05 and ^##^ *p* < 0.01 vs. H_2_O_2;_^§^ *p* < 0.05 vs. corresponding treatment between groups; **c**, control group; **S**, sildenafil. Experiments were repeated at least three times (*n* = 3) with essentially identical results.

**Figure 2:**
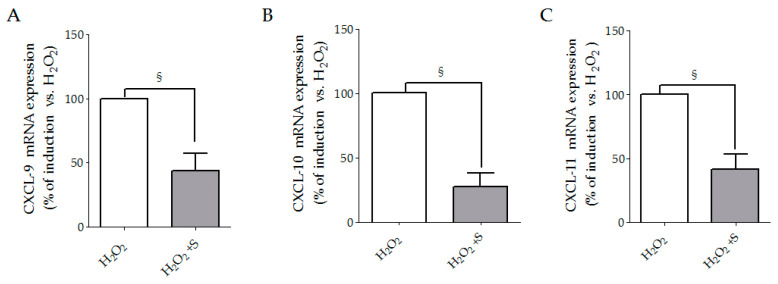
CXCL-9 (**A**), CXCL-10 (**B**) and CXCL-11 (**C**) mRNA of SSc fibroblasts cultures exposed to H_2_O_2_ (100 μM, 24 h) in the presence or absence of sildenafil (1 μM). Data are shown as the fold increase vs. H_2_O_2_ taken as 100% ± SEM (*n* = 3). Statistical significance was determined using the Mann–Whitney *t*-test. ^§^ *p* < 0.05 of H_2_O_2_ vs. H_2_O_2_ + S. **S**, sildenafil. Experiments were repeated at least three times (*n* = 3) with essentially identical results.

In comparison to control cells, H_2_O_2_ treatment induced a strong release of CXCL-9, CXCL-10 and CXCL-11 in SSc fibroblasts respectively by 4.5 ± 1.6, 5.2 ± 1.2 and 5.9 ± 2.4 fold (Figure 1A–C), whereas no effects were observed in healthy cells. Pre-treatment with sildenafil decreased the effects of H_2_O_2_ in SSc cells by 47.2% for CXCL-9, 57.5% for CXCL-10 and 58.9% for CXCL-11 (Figure 1A–C). Since we did not observe differences in healthy controls, we analyzed the mRNA expression for CXCL-9, CXCL-10 and CXCL-11 only in SSc. The experiments of gene expression by real-time PCR showed the ability of sildenafil to reduce, after H_2_O_2_ treatment, the mRNA for CXCL-9 by 55%, for CXCL-10 by 73% and for CXCL-11 by 60% (Figure 2A–C). The presence of the PDE5i alone did not produce any significant change (data not shown).

### 3.2. Sildenafil Impaired the Activation of Intracellular Pathways Underlying Chemokines Expression in SSc Fibroblasts

**Figure 3:**
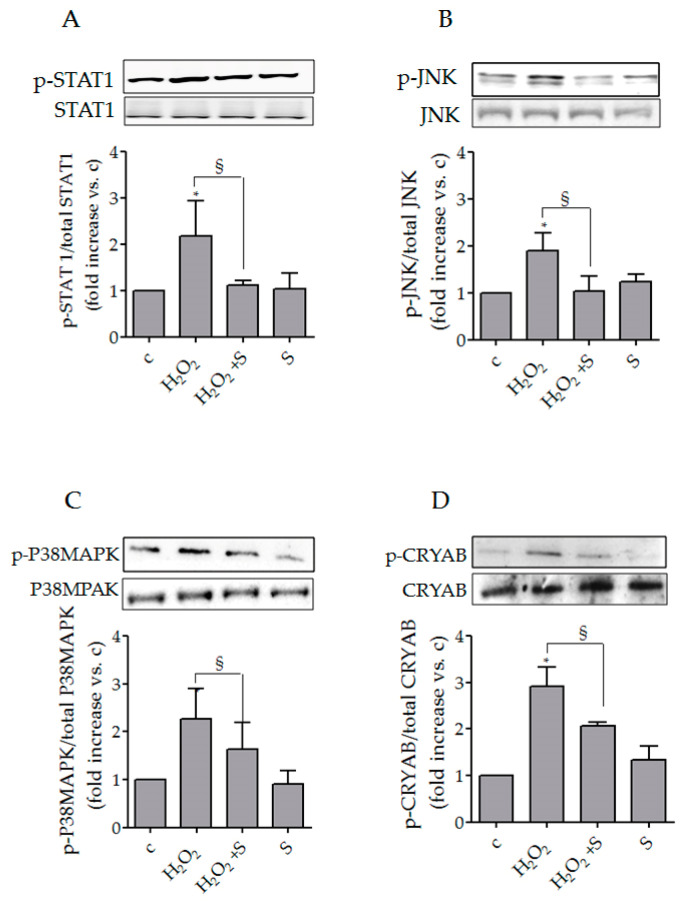
Representative Western blot images of proteins analyzed in SSc fibroblasts. Proteins extracted from SSc fibroblasts exposed to H_2_O_2_ (100 μM) with or without sildenafil (1 μM) were immunoblotted with antibodies against the total and phosphorylated form of STAT1 (**A**), JNK (**B**), P38MAPK (**C**) and CRYAB (**D**). Bars of the histograms show the ratio between the phosphorylated and total forms of the protein targets. Statistical significance was determined using ANOVA with Bonferroni’s post-hoc test. * *p* < 0.05 vs. relative control within group; ^§^ *p* < 0.05. **S**, sildenafil. Experiments were repeated at least three times (*n* = 3) with essentially identical results.

The effects of sildenafil on intracellular signaling molecules well-known to regulate the gene expression of CXCL chemokines, specifically STAT1, P38MAPK and JNK, were investigated in SSc fibroblasts. We observed that the pro-oxidant condition induced an increase in p-STAT1 by 2.2 ± 0.8 (*p* < 0.05), in p-JNK by 1.9 ± 0.4 (*p* < 0.05), and in p-P38MAPK by 2.3 ± 0.7 (*p* < 0.05). Sildenafil treatment impaired the upregulations restoring the p-STAT1, p-JNK and p-P38MAPK phosphorylation similar to the control levels (Figure 3A–C).

### 3.3. Sildenafil Reduced p-CRYAB (Ser59)/CRYAB Induced by Hydrogen Peroxide in SSc Fibroblasts

As expected, the analysis of a stress response protein, CRYAB, normally involved in the response to numerous stimuli (including heat, mechanical and oxidative stress [33,34,35]) has revealed a significant increase in its activation in SSc fibroblasts following exposure to hydrogen peroxide (100 μM, 1 h) (H_2_O_2_: 2.92 ± 0.4 fold change vs. c, *p* < 0.05). Pre-treatment with sildenafil (1 μM) significantly reduced the activated form of CRYAB induced by pro-oxidant (H_2_O_2_ + S: 2.1 ± 0.08 fold change vs. H_2_O_2_, *p* < 0.05). No changes were observed in the presence of only sildenafil (*p* > 0.05) (Figure 3D).

### 3.4. Sildenafil Reduced CXCR3 Plasma Membrane Localization Induced by Hydrogen Peroxide in SSc Fibroblasts

**Figure 4:**
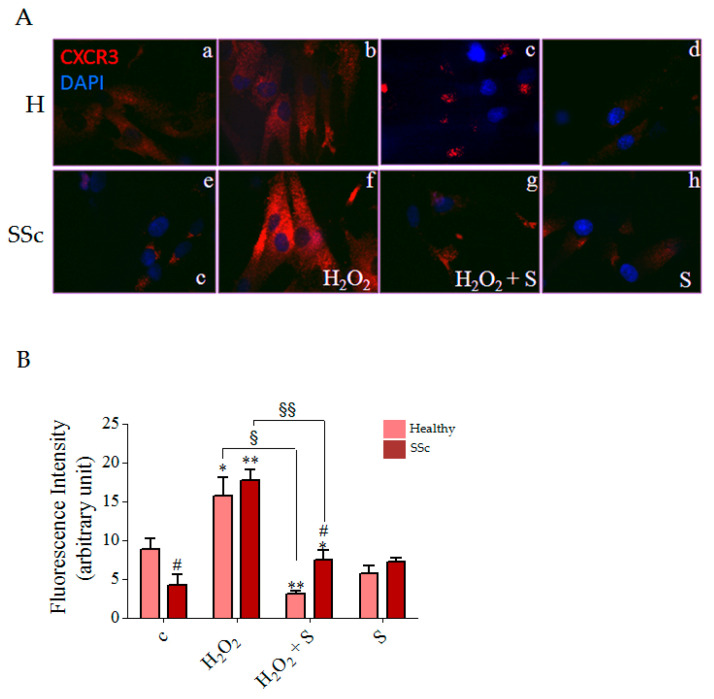
(**A**) Healthy (H) (upper panels) and SSc (lower panels) fibroblasts were exposed to H_2_O_2_ (100 μM, 1 h) in the presence or absence of sildenafil (1 μM) pre-treatment. Fixed non-permeabilized cells were stained for CXCR3 antibody (red) and DAPI for nuclei (blue). (**B**) Histograms represent the fluorescence intensities of healthy (red columns) and SSc (pink columns) fibroblasts for CXCR3 in the plasma membrane. Statistical significance was determined using ANOVA with Bonferroni’s post-hoc test. * *p* < 0.05 and ** *p* < 0.01 vs. relative control within group; ^#^ *p* < 0.05 vs. H_2_O_2;_ ^§^ *p* < 0.05 and ^§§^ *p* < 0.01 vs. corresponding treatment between groups; **c**, control group; **S**, sildenafil. Experiments were repeated at least three times (*n* = 3) with essentially identical results.

To investigate the effect of sildenafil on CXCL-9, -10 and -11 receptor localization in a pro-oxidant condition, we performed an immunofluorescence experiment for CXCR3 on non-permeabilized healthy and SSc fibroblasts treated with 100 μM H_2_O_2_ for 1 h in the presence or absence of sildenafil 1 μM. After treatment with H_2_O_2_, we observed a significant increase in CXCR3 on the plasma membrane in comparison to the basal conditions both in the healthy and SSc cells by 1.8 fold and 4.2 fold respectively (Figure 4A, inserts a,e and b,f; Figure 4B). Pre-treatment with sildenafil significantly reduced the level of CXCR3 by 19.7% in healthy and 42.1% in SSc cells (Figure 4A, inserts c and g, Figure 4B). Interestingly, healthy fibroblasts showed higher CXCR3 localization on plasma membrane than SSc (*p* < 0.05), with a tendency to decrease after sildenafil treatment.

## 4. Discussion

SSc is a complex disease with heterogeneous clinical features and disease severity, corresponding to the extent of skin fibrosis and internal organ involvement. Several lines of evidence indicate that systemic scleroderma presents a deregulated production of cytokines implicated in vascular damage and fibrosis, but their relationship with clinical symptoms is still unclear [2,10]. To this purpose, several studies have been addressed to find biomarkers and new potential pharmacological targets mirroring the progression of the pathological process and predicting disease prognosis and treatment response [8,9,10,12,36].

In this study, we provided evidence that the vasoactive drug sildenafil [37] reduces the expression and secretion of specific chemokines in SSc dermal fibroblasts exposed to ROS by interfering with the pro-inflammatory pathways involved in their expression and release.

In recent years, the participation of local tissue cells such as fibroblasts, cardiomyocytes and myocytes in the pathogenesis of the inflammatory events sustaining allo-driven or autoimmune-driven tissue inflammation and damage has been increasingly recognized [3,38,39,40]. Here, we showed that SSc fibroblasts exposed to a pro-oxidant stimulus, a feature condition of SSc cells [41], produce and secrete a significant amount of the interferon-γ induced CXC chemokines, such as CXCL-9, -10 and -11, compared with healthy fibroblasts. These data confirm that, depending on the type of stimulus, SSc fibroblasts could be an important source for the production of chemokines and therefore contribute to the increased serum levels of these molecules observed in SSc patients [3,42,43].

Chemokines are a large family of small (7–15 kDa), structurally related heparin-binding proteins that may participate in immune and inflammatory responses through the chemo-attraction and activation of leukocytes [44,45]. In SSc, previous studies have suggested a net increase in selected chemokines in the skin and systemically [12]. In particular, it has been observed that serum CXCL-10 levels are increased in pre-clinical (non-fibrotic)/early SSc patients, and its high concentration indicates a faster rate of progression from pre-clinical/early SSc to worse disease stages [46], as well as a possible organ involvement [42]. Of note, we recently demonstrated that CXCL-10 concentration is associated with the worsening of capillaroscopic pattern and reflects disease progression from very early diagnosis of SSc (VEDOSS) to SSc condition [15]. Similarly, the plasma level of CXCL-11 has been suggested as an early indicator of vascular bed alteration/modification/rearrangement [15], and is strictly associated with the severity of lung, skin and muscle involvement [47].

To date, further studies are needed to elucidate the role of CXCL-9 in SSc. However, its levels were found to be significantly elevated in SSc serum and associated with a more severe clinical phenotype involving tissues such as kidney, lung and thyroid [11,41,48,49].

We previously demonstrated in SSc fibroblasts that sildenafil counteracts the negative effect of ROS on cell viability and proliferation, thereby promoting the activity of specific antioxidant enzymes involved in redox homeostasis [26]. In addition, this PDE5i exerted an inhibitory effect on gene expression and release into the culture medium of selected cytokines (IL-6 and IL-8) by interfering with the activation of upstream pro-inflammatory pathways such as STAT3, ERK, PKB/AKT and NFκB [25]. We also found that sildenafil can significantly decrease CXCL-10 protein release by cardiac and endothelial cells [50,51]. Consistent with these findings, here we observed for the first time that the sildenafil inhibited CXCL-9, CXCL-10 and CXCL-11 gene expression and release into the culture medium of SSc fibroblasts exposed to ROS. It remains to be investigated whether this could be the result of persistent exposure to pro-oxidants and/or of the reduced antioxidant capacity/sensitivity of these cells [26,52].

To dissect the potential mechanism by which sildenafil can modulate the expression and secretion of CXCL chemokines, we analyzed the modulation of proteins such as STAT1, JNK and p38MAPK, known to be also involved in ROS-mediated signaling and chemokines secretion [25,50,53,54]; we also analyzed the membrane localization of CXCR3, a chemokine receptor belonging to a subgroup of class A G-protein-coupled receptors specific for CXCL-9, CXCL-10 and CXCL-11 binding and activation [55]. We observed a greater modulation of these molecules with an increase in their activation and/or membrane localization in SSc compared with healthy fibroblasts, supporting the already proposed notion that SSc fibroblasts have a reduced ability to counteract the redox unbalance [6,26]. It is also well known that the increase in ROS is involved in the activation of small heat shock proteins (sHSPs), including CRYAB [35,56]. The biological role of these proteins is mainly to act as “holdases,” interacting with partly or completely unfolded proteins in the early phase of stress response and then facilitating their refolding through the “foldase” activity of other HSPs [57]. As already demonstrated in other studies [33,34], non-cytotoxic concentrations of ROS induce a significant increase in only the phosphorylated form of CRYAB. Interestingly, the presence of sildenafil significantly reduced the phosphorylation levels of all aforementioned proteins, confirming its involvement in ROS-mediated signaling through the modulation of signal transducers, transcription activators and stress response proteins [25,26].

Therefore, we believe that, despite not offering a complete explanation, our results add a new piece to the puzzle that defines the molecular mechanism underlying this novel biological effect of sildenafil. In this sense, it would be worth exploring the extent to which this effect is directly mediated by cyclic nucleotide hydrolysis inhibition or by independently elevating levels of cAMP and cGMP or modulating ion channels in tissue fibroblasts [27].

Finally, to date, there is no approved treatment of sildenafil for skin lesions, digital ulcers and skin thickness, and as such, further results validated in a larger controlled study are needed. Although the lack of positive effects of this molecule on collagen formation and extracellular matrix deposition, parametersassociated with skin thickness in SSc [23,58], our data on SSc fibroblasts support previous reports [21,22,23,24] and encourage its use, alone or in combination with other molecules, as a therapeutic strategy to treat skin fibrosis.

## 5. Conclusions

**Figure 5:**
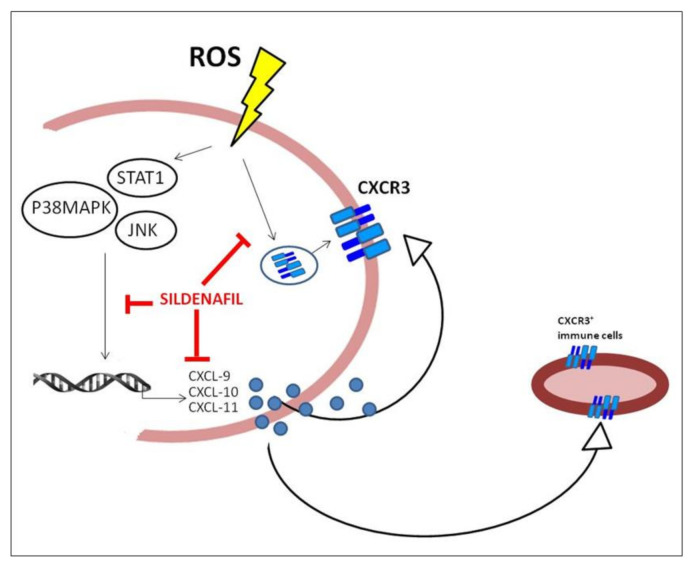
**Suggested mechanism of action of sildenafil on SSc fibroblasts.** After a pro-oxidant stimulus, the cell activates intracellular pathways that rapidly induce both the translocation of the CXCR3 receptor on the plasma membrane and the phosphorylation of downstream signaling molecules such as STAT1, JNK and p38MAPK. These proteins adapt the DNA response inducing the expression and release of the pro-inflammatory chemokines CXCL-9, CXCL-10 and CXCL-11. The binding of these chemokines to the specific receptor CXCR3 on the plasma membrane increases and amplifies the pro-inflammatory response towards autocrine/paracrine action and recruits CXCR3+ immune cells to tissue damage. The action of sildenafil results in an impairment of CXCR3 recruitment on the plasma membrane and in the counteraction of the transduction pathway responsible for chemokines expression and release. This can determine the reduction of the inflammatory activation loop at the site of damage.

In conclusion, our data support the role of fibroblasts themselves as part of the immune system, and not only as structural elements. As recapitulated in Figure 5, we show that a number of chemokines, such as CXCL-9, CXCL-10 and CXCL-11, are produced and released by SSc fibroblasts exposed to ROS, potentially contributing to the pathogenesis of fibrosis. The presence of sildenafil has been demonstrated to counteract the CXCR3 recruitment on the plasma membrane and to inhibit the activation of molecules involved in the transduction pathway responsible for chemokines expression and release.

Nevertheless, although it was conducted in vitro and on a limited set of samples, this analysis highlights the potential therapeutic use of sildenafil extended not only for the treatment of patients at risk of developing SSc but also for designing future therapeutic strategies for vascular damage, immune deregulation and fibrosis in SSc.

## Figures and Tables

**Table 1:** Sequences of primers for RT-PCR analysis.

Gene Name	Forward (5′–3′)	Reverse (5′–3′)
*CXCL-9*	CTGTTCCTGCATCAGCACCAAC	TGAACTCCATTCTTCAGTGTAGCA
*CXCL-10*	TTCCTGCAAGCCAATTTTGT	ATGGCCTTCGATTCTGGATT
*CXCL-11*	GAGTGTGAAGGGCATGGCTA	ACATGGGGAAGCCTTGAACA
*β-ACTIN*	CTGAACCCCAAGGCCAAC	AGCCTGGATAGCAACGTACA

## Data Availability

The data presented in this study are available on request from the corresponding author. The data are not publicly available due to ethical restriction.
